# Mental imagery interventions to promote face covering use among UK university students and employees during the COVID-19 pandemic: study protocol for a randomized controlled trial.

**DOI:** 10.1186/s13063-021-05852-y

**Published:** 2022-01-18

**Authors:** Dominic Conroy

**Affiliations:** School of Social Sciences and Professions, London Metropolitan University, Tower Building, 166-220 Holloway Road, London, N7 8DB UK

**Keywords:** Imagery face coverings, Behaviour, Intervention, Health, COVID-19, Intention, Attitudes, Norms, Control, Self-efficacy

## Abstract

**Background:**

The 2020 COVID-19 pandemic has witnessed wide-ranging efforts to minimize the spread of the virus and to protect those most vulnerable to becoming unwell following viral infection. Core COVID-19 preventive measures include social distancing, regular hand washing, and wearing face coverings in public places. Understanding links between social cognitive factors relating to beliefs/skills is important in the context of the COVID-19 pandemic, as this can suggest which factors might be targeted via behaviour change interventions to promote adherence to COVID-19 preventative behaviours. In this context, mental imagery exercises—self-directed imagining of an anticipated outcome or processes linked to a defined behaviour/activity—offer a well-evidenced, relatively simple behaviour change intervention. In the mental imagery invention reported in this protocol, individuals will be randomly assigned to one of four separate conditions (outcome imagery, process imagery, outcome and process imagery, control).

**Methods:**

The primary objective of this randomized controlled study is to assess the effectiveness of a mental imagery intervention on wearing face coverings, as a defined core COVID-19 preventative behaviour. Participants will consist of UK university students and university employees of any age. Participants will be randomized to complete an ‘outcome imagery’ or a ‘process imagery’ exercise, both exercises (i.e. a combined condition) or neither exercise (i.e. a control condition). A total of 260 individuals will be recruited into the study. Outcomes for all study condition arms will be assessed at baseline (Time 1), immediately post-intervention (Time 2), and at 1-month follow-up (Time 3).

The primary outcome is frequency of wearing face covering, as reported at T2 and T3. Secondary outcomes include intervention effects on face covering attitudes, social norms, perceived behavioural control and barrier self-efficacy at T2 and T3. Putative moderators of intervention effects are conscientiousness, narcissism and ‘light triad’ personality traits.

**Discussion:**

This trial will contribute toward the currently sparse evidence base concerning behaviour change techniques designed to promote COVID-19 preventative behaviours among UK university students and university employees.

**Trial registration:**

ClinicalTrials.gov (U.S. National Library of Medicine) NCT04583449. Retrospectively registered on 20 October 2020.

**Supplementary Information:**

The online version contains supplementary material available at 10.1186/s13063-021-05852-y.

## Administrative information

Note: the numbers in curly brackets in this protocol refer to SPIRIT checklist item numbers. The order of the items has been modified to group similar items (see http://www.equator-network.org/reporting-guidelines/spirit-2013-statement-defining-standard-protocol-items-for-clinical-trials/).

**Table:** 

Title {1}	A randomized controlled mental imagery intervention to promote face covering use among UK university students and employees during the COVID-19 pandemic: Study Protocol
Trial registration {2a and 2b}.	This trial is registered with ClinicalTrials.gov, NCT04583449.
Protocol version {3}	Version 1
Funding {4}	This trial is unfunded.
Author details {5a}	London Metropolitan University, U.K.
Name and contact information for the trial sponsor {5b}	Not applicable as this research is unfunded.
Role of sponsor {5c}	Not applicable as this research is unfunded.

## Introduction

### Background and rationale {6a}

The 2020 COVID-19 pandemic has witnessed wide-ranging efforts to minimize the spread of the virus and to protect those most vulnerable to becoming unwell following viral infection. At the time of preparing this protocol, the three core ‘COVID-19 preventive measures’ included social distancing, regular hand washing and wearing face coverings enclosed public places [1]. Despite scientific debate around the evidence base in support of using face coverings in the context of the COVID-19 virus the scientific consensus overwhelmingly now supports the role of face coverings as a key protective measure pivotal to saving lives during the pandemic [2–4]. The current scientific consensus is that face coverings are an important mechanism for protecting others than face covering wearers themselves, and that even limited wearing of face coverings among the general public is likely to decrease viral transmissibility and, therefore, will save lives [5–7]. The UK government made using face coverings in defined indoor public areas (e.g. on public transport, in shops/supermarkets, in places of worship) a legal requirement in July 2020 and has been enforced with potentially substantial fines (up to £6400/$8700) [8].

Behavioural science has already guided understanding of how COVID-19 preventative measures, including face covering adherence, may be predicted. For example, evidence from varied continental settings has suggested that threat perceptions may predict adherence to COVID-19 preventive behaviours [9–12]. Given that face coverings primarily offer protection to individuals other than the wearer, individual threat perceptions and psychological theories concerned with risk susceptibility/vulnerability (e.g. Protection Motivation Theory; the Health Belief Model) may have less predictive utility in the context of the 2020 pandemic. Social-cognitive models have been tested to explain how variability in core beliefs relating to wearing face coverings might predict face covering adherence. For example, measuring whether individual’s hold (un)favourable beliefs about wearing face coverings (i.e. attitudes); perceived beliefs held by important others (e.g. friends and family) about wearing face coverings; or an individual’s perceived behavioural control or self-efficacy in the context of wearing face coverings [13, 14]. Various integrated social cognition models have been assessed as frameworks for predicting COVID-19 preventive behaviours [15–17]. For example, evidence has suggested that greater levels of preparedness to avoid foreseeable obstacles linked to a specific behaviour are predictive of increased COVID-19 preventative behaviour adherence at 1-week follow-up [15].

Understanding links between social cognitive factors relating to beliefs/skills is important in the context of the COVID-19 pandemic, as this can suggest which factors might be targeted via behaviour change interventions to promote adherence to COVID-19 preventative behaviours. At the time of writing few studies have tested interventions designed to elicit behavioural change in the context of COVID-19 preventative behaviours. Unpublished evidence revealed no difference in the impact of five health communication messages each of which emphasized a particular psychological mechanism involved in modifying the likelihood of adhering to COVID-19 preventative behaviours (e.g. self-protection, ‘sunk costs’, i.e. energies already invested in fighting the pandemic; disgust at non-adherent behaviour or easing cognitive load, i.e. message simplification) [18]. Given this paucity of empirical research exploring behaviour change interventions, despite the clear role for behavioural science in the context of COVID-19 preventative behaviour, it will be of interest to test the efficacy of a mental imagery behaviour change intervention. Mental imagery exercises are well evidenced, relatively simple behaviour change interventions in the context of behaviours relevant to promoting physical health and have been described by Conroy and Hagger as ‘self-directed imagining or visualizing specific events, actions or outcomes, including concomitant feelings and responses, with the express purpose of increasing motivation toward a target action or task’ (p. 669) [19]. Mental imagery interventions involve exercises requiring visualization and may also involve a written component relating to visualized health-related action. Mental imagery exercises can involve focusing on anticipated positive/beneficial outcomes of an action (outcome imagery) or imagery relating to the anticipated strategies/preparation that would be required to successfully execute a pre-defined action (process imagery).

While encouraging adherence to subsequent behaviour is the gold standard by which behaviour change interventions should be assessed, interventions can also be assessed in terms of their proven capacity to modify social cognitive ‘proxies’ of behaviour including those defined above (e.g. attitudes towards face coverings and skills involved in successfully wearing face coverings where required). In addition to belief/skill-based factors, variability in an individual’s adherence to wearing face coverings varies in association with what ‘type’ of person that individual is in dispositional terms will also be of interest. For example, a relatively conscientious person [20] might be expected to be more likely to routinely wear a face covering to protect nearby others from the risk of viral infection. Accordingly, personality traits relevant to COVID-19 preventative behaviours including ‘the light triad’ of Kantianism (i.e. treating people as valued in and of themselves), Humanism (i.e. valuing the dignity and worth of individuals) and Faith in humanity (i.e. beliefs that people are fundamentally good) [21] and conscientious, described above, are of interest. However, choosing to wear a face covering (or not) might vary in association with an individual’s vanity/egotistical self-image and therefore we will also assess whether/how intervention effects vary in association with narcissism [22]. Many mental imagery interventions employ measures of individual differences in dispositional skills or practice with using imagery relating to a defined behaviour [23]. Evidence has suggested that variability in skill/ability with visualizing (or, ‘imagery ability’) may modify the effectiveness of imagery intervention effectiveness (e.g. [24]). Imagery ability can, therefore, be a useful variable to gauge when testing imagery interventions.

This paper presents the study protocol for a three-part imagery intervention designed to encourage adherence to COVID-19 preventative behaviours. Participants are prompted to engage in the following three steps. In part 1, participants will be provided with information about the defined COVID-19 preventative target behaviour (i.e. wearing a face covering) as applied to a UK cultural context. In part 2, participants will be asked to visualize themselves wearing a face covering (the precise emphasis of this imagery exercise will vary depending on the study condition). In part 3, participants will be requested to record specific components derived from completing this imagery exercise (again, precise emphasis depending on condition) in written form.

### Objectives {7}

#### Goals and objectives

Drawing on recent meta-analytic conclusions which have substantiated the value of mental imagery interventions in the context of health behaviours [19] and a template approach for preparing rigorously developed, well designed mental imagery interventions [25], we planned to develop a theory-based behavioural intervention to promote face covering adherence, and more favourable social cognitions toward face covering adherence, among UK university employees and students. Individuals recruited will be randomly assigned to one of four separate conditions (outcome imagery, process imagery, both outcome and process imagery, control). Participants assigned to an imagery condition will be provided with information about the defined COVID-19 preventative target behaviour; will be asked to visualize themselves wearing a face covering; and will be requested to record specific features of imagery generated during the imagery exercise in written form. The control condition will receive a health warning message drawn from a pool of standard/representative UK government messages alone (representing a ‘usual care’ face covering warning control condition). Study measures will be taken at three time points: at baseline prior to randomization (i.e. Time 1, T1 hereafter); immediately post intervention (i.e. Time 2, T2 hereafter); and at 1-month follow-up (i.e. Time 3, T3 hereafter). The time interval between T1 and T2 will vary depending on intervention condition but will last approximately 15–20 min. The primary goal of this randomized controlled trial (RCT) is to evaluate the efficacy of a mental imagery intervention designed to promote face covering adherence on face covering behaviour at T3. The secondary goal of this RCT is to evaluate the efficacy of the imagery intervention on social cognitions at T2 and T3.

##### Specific objectives

1. To assess the impact of mental imagery interventions relative to a control condition comparing baseline measures (T1) with measures taken immediately post intervention (T2) and at 1-month follow-up (T3) relative to T1.
2. To assess more favourable social cognitive indicators of face covering adherence at T2 relative to T1 and at T3 relative to T1.
3. To assess relevant social cognitive factors (e.g. attitudes, normative beliefs, self-efficacy) as mediators of intervention effects on behaviour and cognitions at T2 and T3 (relative to T1).
4. To examine personality traits as moderators of intervention effects at T3.
5. To explore variability in imagery ability (how capable individuals are at visualizing future actions) as a moderator of intervention effects at T3.

##### Hypotheses

It is hypothesized that individuals assigned to any imagery intervention condition will report, at T2 and T3:

*H1*. Significantly higher levels of T2 behaviour (i.e. self-reported face covering adherence) relative to the control condition.

*H2*. Significantly higher intentions to wear face coverings in public places where these are required, relative to the control condition.

*H3*. Significantly more favourable attitudes, subjective norms, barrier self-efficacy and perceived behavioural control linked to face covering wearing relative to control condition participants.

*H4*. Finally, that imagery intervention effects will be conditional on being more conscientious, less narcissistic, and being characterized by higher levels of light triad personality traits.

### Trial design {8}

#### Methods/design

**Fig. 1:**
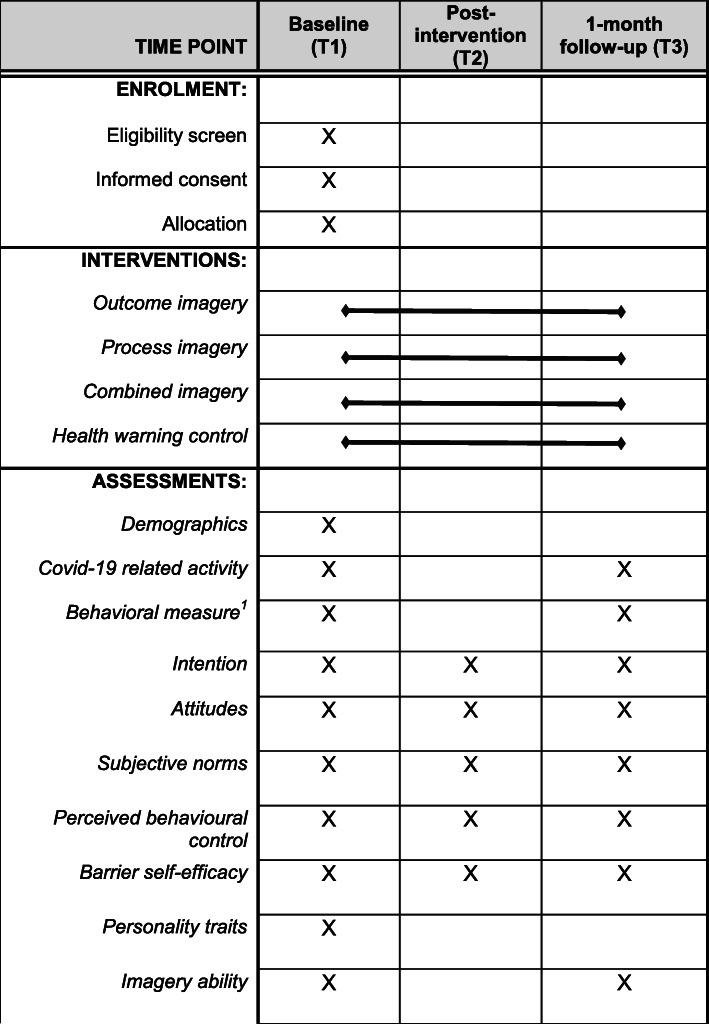
Schedule* of enrolment, interventions, and assessments for trial of mental imagery to improve adherence to face covering wearing adherence. *Adapted from SPIRIT Checklist guidance material displayed online under ‘Standard Protocol Items. Recommendations for Interventional Trials (SPIRIT)’. Self-reported face covering adherence for the previous week

This study protocol is reported in accordance with Standard Protocol Items: Recommendations for Interventional Trials (SPIRIT) standard protocol items for clinical trials [26, 27]. For full details of the schedule of enrolment, interventions, and assessments for the planned intervention study, please consult Fig. 1.

#### Design

An experimental, prospective design will be adopted. Participants will be randomized to one of four conditions (outcome imagery; process imagery; outcome and process imagery; control ‘face covering warning’ condition). Mental imagery interventions will be evaluated against the control condition immediately post-intervention (T2) and at 1-week follow-up (T3). The evaluation will adopt a 2 (outcome imagery vs no outcome imagery) × 2 (process imagery vs no process imagery) × 3 (time: T1, T2, T3).

## Methods: Participants, interventions and outcomes

### Study setting {9}

#### Setting

This study will be conducted from two UK universities based in England. Given the social distancing restrictions following the COVID-19 pandemic, all measures will be gauged via Qualtrics, an online data collection interface (https://uelpsych.eu.qualtrics.com). There were divergences in the timing, duration, and stringency of their national responses by all four nations of the UK (England, Scotland, Wales and Northern Ireland) including formal evidence, for example, that Scotland took a relatively stringent approach to managing the pandemic relative to other UK nations [28]. Despite these differences, broad consistency in public health measures and economic support taken across UK nations has also been acknowledged [28]. Restricting focus to UK nations is important given that government guidelines and restrictions linked to the pandemic as well as the trajectory of the pandemic itself can diverge more considerably beyond UK nation strategic decisions. The UK has suffered relatively badly during the pandemic with, at the time of writing, over 8.2 million confirmed cases of COVID-19, over 550,000 COVID-19 related hospitalizations and over 137,000 deaths associated with COVID-19 recorded since 31st January 2020 [29–31]. Therefore, restricting the focus of the intervention reported in this article to the UK alone, as a national setting on which the impact of the pandemic has been relatively severe, is warranted.

#### Participants

A convenience sampling approach will involve recruiting adults aged 18 years or older who either study or work at one of two UK Higher Education institutions. Our eligibility/selection criteria will require participants to complete questionnaires at both time points; to be UK residents; to engage to a satisfactory level with intervention exercises; and to have regular exposure to circumstances/situations where wearing face coverings would be required under UK law. A focus on privileging the recruitment of individuals affiliated with university campuses (either as students or employees) is arguably justified given that (in circumstances of eased restrictions) travel to campuses, and social mixing in campus spaces would hold clear risks in terms of virus transmissibility. In reports of the intervention study, we will include details concerning the extent to which our final sample will be representative of the demographic characteristics found in the broader UK general population (e.g. in terms of age, sex, socioeconomic status). Full details of what study participation will involve in terms of activities and time requirements will be provided on the first page of the Qualtrics survey. Where individuals reading these details are willing to participate in the study, they will be able to provide their informed consent using a checkbox on the bottom of the first survey page.

### Eligibility criteria {10}

- UK resident at time of completing questionnaires at all time points (T1, T2, T3)
- Completion of the full questionnaire at time points
- Clear evidence of engagement with the mental imagery exercise (all responses will be screened and unclear/irrelevant or indifferent responses will be excluded from analysis).
- Regular (minimum of 2–3 times per week) exposure to indoor public places where face coverings would be required (under UK law)
- Provision of informed consent

### Who will take informed consent? {26a}

SPIRIT guidance: Who will obtain informed consent or assent from potential trial participants or authorized surrogates, and how (see Item 32). Details are provided in the ‘Study setting {9}’ section above.

### Additional consent provisions for collection and use of participant data and biological specimens {26b}

Not applicable as no additional consent provisions for collection and use of participation data and biological specimens will be collected as part of this trial.

## Interventions

### Explanation for the choice of comparators {6b}

Outcome and process imagery exercises will be chosen as the standard variants used in mental imagery intervention studies applied to physical health behaviours. A combination condition (i.e. outcome and process exercises presented in sequence) will be included to test (a) whether synergies between imagery exercises affected study outcomes and (b) to assess effects of intervention length on study outcomes. The health message control condition will be included on the basis that this is the standard visual/verbal health promotion warning encountered by UK citizens during the pandemic from July 2020 to the time of writing this protocol.

### Intervention description {11a}

The mental imagery intervention described in this protocol consists of two distinct imagery exercises—an ‘outcome imagery’ exercise and a ‘process imagery’ exercise. The complete text for the imagery intervention imagery conditions is included as an online supplementary resource. All imagery materials and written exercises will be hosted via an online questionnaire. Participants will be randomized to one of four conditions (outcome imagery; process imagery; outcome and process imagery; control ‘face covering warning’ condition). Each participant randomized to one of the three imagery exercise conditions will engage in an imagery exercise presented in three sequential parts. In part 1, participants will receive information about wearing face coverings in indoor public places. Specifically, imagery condition participants will read the following text defining face covering use in the context of the COVID-19 pandemic: ‘In the context of the coronavirus (COVID-19) outbreak, a face covering is something which safely covers the nose and mouth. You can buy reusable or single-use face coverings. You may also use a scarf, bandana, religious garment or hand-made cloth covering but these must securely fit round the side of the face’. In part 2, participants will be asked to visualize themselves wearing a face covering where this is required by UK law (i.e. in indoor public places). The importance of imagining distinctive, personally relevant visual imagery will be underscored in the passage providing instructions to all imagery condition participants. In part 3, participants will be asked to write about the mental images associated with the imagery exercise that they have completed. As detailed above, completion of imagery exercises is estimated to take approximately 8–12 min in total for single imagery interventions and between 15 and 25 min in total for combined imagery condition participants (i.e. those individuals completing both outcome and process imagery exercises).

#### Outcome imagery condition

Outcome imagery condition participants will be asked to visualize themselves successfully wearing a face covering in all required public places/situations over the coming week, and to imagine how they would feel (i.e. positive outcomes of having successfully worn face coverings where required to do so). Outcome imagery participants will then be asked to write in a free-text box how they would feel having successfully worn a face covering in required indoor public places/situations over the week ahead.

#### Process imagery condition

Process imagery condition participants will be asked to visualize the kinds of strategies involved in successfully wearing a face covering in all required public places/situations over the coming week. Process imagery participants will then be asked to write in a free-text box about the kinds of strategies that would be involved in successfully wearing a face covering in all required indoor public places/situations over the coming week.

#### Combined imagery condition

A third experimental condition will receive both outcome and process imagery exercises to read and complete in sequential order. The sequence will be ‘outcome exercise’ followed by ‘process imagery’ for all combined imagery condition participants. Following this sequence for all participants in this condition is based on the rationale that to imagine factors involved in successfully wearing face coverings where required it is intuitive to have first reflected on potential advantages/positive outcomes of doing so.

#### Control condition

A fourth condition will involve viewing a UK Government public health message circulated on social media as an image concerning the importance of wearing face covering while in public places. This message will be selected as a representative online message available on social media (https://twitter.com/GOVUK/status/1286304635767140352) in early August 2020 at which time the intervention was under development. The image is representative of UK Government public health guidance on wearing face coverings in public places during the COVID-19 pandemic at this time [8].

### Criteria for discontinuing or modifying allocated interventions {11b}

Not applicable as the intervention set-up did not require the facility for participants to be discontinued or for allocated interventions to be modified as part of this trial.

### Strategies to improve adherence to interventions {11c}

Not applicable as no strategies to improve adherence to interventions will be included as part of this trial.

### Relevant concomitant care permitted or prohibited during the trial {11d}

Not applicable as variability in permitted/prohibited concomitant care, to the extent that it meaningfully applies to this intervention, will not be monitored as part of this trial.

### Provisions for post-trial care {30}

Not applicable as provisions for post-trial care will be made as part of this trial.

### Outcomes {12}

Study outcome measures are shown in Table 1. Behavioural outcome data will be recorded at T1 and T3 and psychological outcome data will be recorded at T1, T2 and T3 (see Fig. 1 for full details of the schedule of assessments). The primary outcome measure is self-reported face covering behaviour recorded at 1-month follow-up (i.e. T3). The secondary outcome measures include face covering intention, attitude, subjective norms, perceived behavioural control and barrier self-efficacy (T2, T3).

**Table 1:** Study outcome measures^1^

Domain/construct	Tool^2^	Item(s)	Description
*Primary outcome*			
Behaviour^3^ (face covering adherence) recorded at T3 (i.e. 1-month follow-up)	Self-report measure using item adapted from Fisher et al. (2020)	1	Behavioural gauge of frequency of adherence to wearing face coverings where required.
*Secondary outcomes*			
Intention^4^	Self-report Likert measure items adapted from Theory of Planned Behavior Scale Construction Guidelines reported by Ajzen (2006)	3	Statement of an individual’s motivations/ plans to engage in the target behaviour.
Attitudes^4^		3	Statement of an individual’s beliefs/ endorsement of engaging in the target behaviour.
Subjective norms^4^		1	Statement of an individual’s perceived beliefs relating to important other peoples’ views about their engagement in the target behaviour.
Perceived behavioural control^4^		3	Statement of an individual’s perceived ability to successfully engage in the target behaviour.
Barrier self-efficacy^4^	Self-report measure using items adapted from Hamilton et al. (2019)	6	Statement of an individual’s perceived ability to successfully engage in the target behaviour when faced with obstacles.

^1^Please note that study outcomes are presented following the sequence in which they will be represented in the intervention study itself

^2^All constructs will be measured at T1 (baseline), T2 (immediately post intervention) and at T3 (1-month follow-up) except behaviour which will be measured at T1 and T3 only

^3^Timeframe = the previous week

^4^Timescale = the forthcoming week

#### Behavioural measure

The complete text for the behaviour and psychological measures is included as an online supplementary resource. Self-reported face covering adherence will be measured using a single item based on previously published work [32], adapted to a UK policy/guidelines context. Participants will read the following text: ‘In the past week, when you have gone outside your home for work, grocery shopping, or other activities that involved using public transport, visiting shops/supermarkets, being in enclosed public spaces where social distancing may be difficult, or being in public spaces where you come into contact with people you do not normally meet, how often did you wear a cloth face covering that covered your nose and mouth?’. Participants will provide a response using response items ranging from ‘1’ (never) to ‘5’ (always). This measure of behaviour will be recorded at T1 and T3 only.

#### Psychological measures

Participants will complete belief- and skill-based measures derived from two social-cognitive theories: social cognitive theory [32] and the theory of planned behaviour [33]. These theories were appropriate guiding frameworks given their centrality to behavioural science research conducted in a public health context to date [34, 35] as well as their proven validity in application to viral pandemics as a discrete public health issue [36, 37]. All constructs will be measured on multi-item scales derived based on standard psychometric guidelines [38]. All self-reported psychological measures will be gauged at T1, T2 and T3. Definitions will be provided in scales where required including definitions of ‘face coverings’ (‘In the context of the coronavirus (COVID-19) outbreak, a face covering is something which safely covers the nose and mouth. You can buy reusable or single-use face coverings. You may also use a scarf, bandana, religious garment or hand-made cloth covering but these must securely fit round the side of the face’) and ‘public spaces where this is required’ (‘on public transport; in shops and supermarkets; in enclosed public spaces where social distancing may be difficult; in public spaces where you come into contact with people you do not normally meet’).

##### Intention

Intention to wear face coverings where required will be measured by presenting participants with the stem ‘Over the next week, wearing a face covering while in public spaces where this is required is something...’ followed by three items (e.g. I intend to do). Answers will be provided using response items ranging from ‘1’ (strongly disagree) to ‘5’ (strongly agree).

##### Attitudes

Attitude towards face covering behaviour will be measured by presenting participants with the stem ‘Wearing a face covering while in public spaces where this is required over the next week is something…’ followed by three pairs of response anchors (e.g. ranging from ‘1’ (not worthwhile) to ‘5’ (worthwhile)).

##### Subjective norms

Subjective norms will be measured using four items in total. This scale will comprise two injunctive norm items (e.g. ‘Most people who are important to me (e.g. friends, family) would want me to wear a face covering while in public spaces where this is required over the next week’); and two descriptive norm items (e.g. ‘Most people who are important to me (e.g. friends, family) will be wearing a face covering while in public spaces where this is required over the next week’). Answers will be provided using response items ranging from ‘1’ (strongly disagree) to ‘5’ (strongly agree).

##### Perceived behavioural control

Perceived behavioural control (PBC) towards face covering behaviour will be measured by three items in total. For one item, participants with the stem ‘How much personal control do you think you have in wearing a face covering while in public spaces where this is required over the next week’ (1 = no control at all, 5 = complete control). For two further items, participants will read a stem statement ‘For me to wear a face covering while in public spaces where this is required over the next week is’ followed by two response options (e.g. ranging from ‘1’ (impossible) to ‘5’ (possible)).

##### Barrier self-efficacy

Barrier self-efficacy towards face covering behaviour will be adapted from a 6-item scale reported in previous work [39] and in line with self-efficacy scale construction guidelines [40]. Participants will respond to the stem ‘Rate your degree of confidence in wearing a face covering while in public spaces where this is required under the following conditions’ followed by six response items (e.g. ‘When I feel stressed/frustrated’; ‘When accessing my face covering is difficult’) and using response options ranging from ‘1’ (cannot do at all) to ‘5’ (highly certain can do’).

#### Personality traits

Personality trait measures will be taken at T1 and examined as moderators of intervention effects. Preceding all personality items, participants will read the stem ‘Describe yourself as you generally are now, not as you wish to be in the future. Describe yourself as you honestly see yourself, in relation to other people you know of the same sex as you are, and roughly your same age’. Participants will then complete scale-specific items as described below.

##### Conscientiousness

Conscientiousness will be measured using a validated scale [20]. Participants will read a further stem ‘Typically, I:’, after which they will provide responses to ten items (e.g. ‘Am always prepared’) including four reverse scored items (e.g. ‘Make a mess of things’). Answers will be provided using response items ranging from ‘1’ (very inaccurate of me) to ‘5’ (very accurate of me).

##### Narcissism

Narcissism will be measured using a scale previously reported elsewhere [22]. Participants will provide responses to 16 items (e.g. ‘People always seem to recognize my authority’). Answers will be provided using response items ranging from ‘1’ (very untrue of me) to ‘5’ (very true of me).

##### Light triad personality traits

Light triads personality traits will be measured using scale reported previously [21]. Participants will complete 12 items in total (4 per trait) relating to Faith in humanity (e.g. ‘I think people are mostly good’); to Kantianism (e.g. ‘I prefer honesty over charm’) and to Humanism (e.g. ‘I tend to treat others as valuable’). Answers will be provided using response items ranging from ‘1’ (very inaccurate of me) to ‘5’ (very accurate of me).

#### Covariates

All covariates will be measured at T1 and T3 only.

##### Demographic measures and COVID-19 related activity

Demographic measures will be collected at T1 including: (i) sex (0=male, 1=female, free text box for other sex); (ii) age (recorded in years); (iii) ethnicity (free text box for self-identified ethnicity); and (iv) occupation (free text box for occupation-related information). An additional measure will be included to approximate time spent in public spaces where COVID-19 protective behaviours would be required by UK law. This measure will consist of three items (e.g. I use public transport on a daily basis). Answers to this measure will be provided at T1 and T3 using response items ranging from ‘1’ (very inaccurate of me) to ‘5’ (very accurate of me).

##### Imagery ability

Dispositional differences in Imagery ability will be measured using an ‘imagery fidelity’ measure described elsewhere [41]. Participants will read the stem statement: ‘When I think about wearing a face covering while in public spaces where this is required over the next week the imagery around this that occurs to me is:’ and will then provide responses using four response items ranging from ‘1’ (not clear) to ‘5’ (clear).

#### Data quality

Three questions will be included in the T1 survey to identify cases of extreme inattentive responding. Evidence has suggested that inattentive responding holds diverse negative implications for data quality (e.g. experimental manipulations, statistical power) and can serve to mask intervention effects. Screening out unambiguously inattentive participant responses may therefore serve to mitigate reduced power and effect size estimation [42, 43]. These items will be set up so that a correct response would only be possible following careful reading of the item (e.g. ‘please choose option four to ensure you are paying attention’). Participants who provided an incorrect response to *all* three items will be excluded from the final dataset. These items will be included as a crude metric for demonstrating protocol adherence (i.e. evidence of a reasonable level of engagement with survey completion) and as a way of identifying where clear non-engagement with the survey is apparent (e.g. the incorrect option chosen for all three responses).

To further maximize data quality, we will omit responses where there is negligible evidence of engagement in the mental imagery exercise (i.e. screened free-text responses that contain unclear/irrelevant or indifferent responses will be excluded from analysis).

### Participant timeline {13}

[The participant timeline is reflected in the SPIRIT diagram included as Fig. 1 in this submission]

### Sample size {14}

#### Statistical power and sample size

An a priori power analysis has been conducted using G*Power V.3.1 for an ANCOVA model estimating fixed effects, main effects and interactions [44]. A medium effect size (*f*=0.25) with power set to 0.80 and alpha set to 0.01 (adjusted given multiple tests and therefore to control for type I error rate inflation) will be sought. Inclusion of six covariates (gender; age; ethnicity; occupation; approximate time spent in places where wearing face coverings would be mandatory; imagery ability) was specified. Power analysis indicated that a minimum sample size of 254 will be required. In developing the recruitment strategy, it is important to be mindful of introducing attrition bias, i.e. bias created by retaining participants with particular characteristics in a way that alters the complexion of the sample. Guidance on acceptable attrition levels has indicated that > 20% study attrition increases the risk of attrition causing serious threats to study validity [45] though debate surrounds precisely when attrition bias may most threaten study validity (e.g. [46, 47]). The study aim is, therefore, to recruit 312 individuals at baseline/T1 to meet a required sample size of 260 at follow-up/T3 (i.e. allowing for no more than 20% attrition).

### Recruitment {15}

#### Enrolment

Details of the schedule of enrolment, interventions, and assessments are shown diagrammatically in Fig. 1.

As described above; all measures will be acquired via Qualtrics, an online platform which will host our study questionnaire. A URL link to the Qualtrics hosted survey will be embedded into an email message promoting the study and this promotional message will be sent out in an email circular message to prospective participants (staff and students at UEL and Manchester University). We will follow bi-weekly enrolment drives to maximize the possibility of an even spread of responses throughout the study recruitment phase, and to maintain a consistent promotional presence of the recruitment message. We will aim to complete all study enrolment within six months: recruitment will start in approximately mid-September 2020 and we will aim to complete all study enrolment (i.e. responses at T1, T2 and T3) by late February 2021. Where enrolment continues for longer than 6 months, we will explore ‘date of participant response’ as a covariate in main intervention study analyses. We will supplement our university-focused recruitment approach by also posting a URL link to the Qualtrics-hosted survey twice each week on social media (e.g. on Twitter, via Facebook posts). Participant recruitment will be led by the protocol author (the UEL-based study researcher) and will be supplemented by recruitment activities led by a second researcher based at Manchester University.

## Assignment of interventions: allocation

### Sequence generation {16a}

After participants have completed complete demographic, psychological and behavioural measures at baseline (T1) they will be randomized to the outcome imagery exercise, the process imagery exercise, a combined condition (comprising the outcome and process imagery exercises) or a control condition. The allocation ratio (i.e. the ratio of participant numbers in each of the conditions) is intended to be equal across groups (i.e. 1:1:1:1). Throughout the recruitment period, the protocol author will conduct biweekly checks to audit that roughly equal allocation of participants to conditions has occurred. Randomization will be conducted via a Qualtrics randomizer features embedded in the T1 survey. The Qualtrics randomizer uses a Mersenne Twister pseudorandom number generator approach which is a widely used approach employed within standard statistical packages (e.g. IBM SPSS, Microsoft Excel). This approach follows a random method by which the randomization sequence cannot be determined until participant assignment has occurred while ensuring roughly even distribution of participants across groups. The researcher team will be blind to group assignment at the point of randomization.

### Concealment mechanism {16b}

Details are provided in the ‘Sequence generation {16a}’ section above.

### Implementation {16c}

Details are provided in the ‘Sequence generation {16a}’ section above.

## Assignment of interventions: Blinding

Details are provided in the ‘Sequence generation {16a}’ section above.

### Who will be blinded {17a}

Details are provided in the ‘Sequence generation {16a}’ section above.

### Procedure for unblinding if needed {17b}

Not applicable as unblinding is not anticipated as a required process within this trial.

## Data collection and management

### Plans for assessment and collection of outcomes {18a}

#### Data collection and management

Participant names will not be included in the dataset and each participant will be assigned a study ID. All data will be downloaded in secure form to institutional cloud services (i.e. OneDrive). All data will be backed-up securely and all data files will be securely password protected and encrypted. Throughout project activities, data files and printed materials that are no longer required will be destroyed. Email address data will be stored in a separate document in an encrypted folder and will be destroyed once recruitment is completed. Range and consistency checks will be conducted on all questionnaire data promptly by the research team. Because questionnaire data will be collected using a ‘Force Response’ setting on Qualtrics there will be no missing data for this study. To address incomplete participant responses we will conduct an intention-to-treat (ITT) analysis in addition to the complete case analysis. ITT is an assessment of all trial participants in analysis regardless of whether or not they provided incomplete responses or failed to provide follow-up responses and provides a way of producing an unbiased estimate of intervention efficacy of the intervention in respect of study adherence [48].

#### Ethics and registration

Ethical approval for this research has been granted from University of East London (UEL) Research Ethics Committee on 13 August 2020 (ID: ETH2021-0006) and from Manchester University Research Ethics committee on 8 September 2020 (UEL approval accepted in lieu of separate institutional approval). Ethical approval involved producing a Data Management Plan which outlines details and procedures for maintaining participant details confidential, and for secure storage and disposal of study data. The trial design process has been methodical, involving iterative stages of development and peer review commentary feedback and, as a result, no protocol modifications are anticipated. However, should protocol amendments be required, institutional ethical amendments will be submitted promptly, and associated trial documentation amended as required in all locations.

### Plans to promote participant retention and complete follow-up {18b}

Not applicable as plans to promote participant retention and complete follow-up will not be included in this trial.

### Data management {19}

Details are provided in the ‘Plans for assessment and collection of outcomes {18a}’ section above.

### Confidentiality {27}

Details are provided in the ‘Plans for assessment and collection of outcomes {18a} section above.

### Plans for collection, laboratory evaluation and storage of biological specimens for genetic or molecular analysis in this trial/future use {33}

Not applicable as no biological specimens will not be collected as part of this trial.

## Statistical methods

### Statistical methods for primary and secondary outcomes {20a}

#### Planned statistical analyses

Across T1–T3, Mean/SD values will be reported for behaviour and the psychological variables and percentages (of total sample) will be reported for demographic variables. Hypotheses stated previously will be tested via a series of ANCOVAs. Intervention condition will be a between-participants variable (outcome, process, combined, control) and time will be a within-participants variable (T1, T2, T3). The primary outcome (face covering behaviour) and secondary outcomes (intention, attitude, subjective norms, PBC, barrier self-efficacy) will be assessed as separate dependent variables. A factorial superiority framework will be adopted for this trial. This approach is preferred to a parallel group trial because it will permit testing for superiority of assignment to a specific imagery exercise technique (e.g. outcome imagery), regardless of study condition, rather than comparing simple assignment to one of the four conditions. Consistent with previous mental imagery interventions, demographic variables will be included as covariates. Where statistically significant effects are demonstrated from ANCOVA analyses, simple effects analyses will be conducted from which estimated marginal means will be reported for all primary and secondary outcome variables. The alpha level will be set at 0.01 for all analyses to control for Type I error rates. Moderation effects of personality traits (conscientiousness, narcissism, Faith in humanity, Kantianism, Humanism) on study intervention effects will be assessed using the PROCESS software macros (V.3.4) on IBM SPSS (V.26).

### Interim analyses {21b}

Because ethical concerns linked to trial participation are not anticipated, and to minimize the Type I error inflation rate, no formal stopping rules or interim analyses are scheduled. However, sub-group analyses will be conducted as prompted by data trends and characteristics (e.g. exploring predictors of non-adherence to wearing face coverings among relevant individuals) or among specific ethnic minority subgroups (e.g. in the UK, Black and South Asian individuals) who may have been disproportionately affected by the COVID-19 pandemic [49].

### Methods for additional analyses (e.g. subgroup analyses) {20b}

Details are provided in the ‘Statistical methods for primary and secondary outcomes {20a}’ section above.

### Methods in analysis to handle protocol non-adherence and any statistical methods to handle missing data {20c}

Details are provided in the ‘Statistical methods for primary and secondary outcomes {20a}’ section above.

### Plans to give access to the full protocol, participant level-data and statistical code {31c}

A public-facing study protocol is available for this study: ‘Mental Imagery to Increase Face Covering Use in UK-based Public Places During the COVID-19 Pandemic’ (ClinicalTrials.gov ID: NCT04583449). Once data collection is completed, the study protocol will be updated with any changes and supplementary files will be added making a participant-level dataset and statistical code (i.e. SPSS syntax files) available to the research community.

## Oversight and monitoring

### Composition of the coordinating centre and trial steering committee {5d}

The primary coordinating centre for the duration of the trial will be UEL where ethical approval for the trial has been secured. Additional research activities will be conducted at a second site (Manchester University) and these activities will be coordinated via UEL. The trial steering committee consists of the protocol author (based at UEL) and the two additional researchers (based at Manchester University).

### Composition of the data monitoring committee, its role and reporting structure {21a}

The data monitoring committee is composed of a single individual based in Library, Archiving and Learning Services at the protocol author’s institution (UEL) at the time of writing this protocol. From the point of study design onwards, the data monitoring committee’s role will be to advise on, and act as a point of liaison for, researcher responsibilities concerning data generation, secure storage of data and correct protocols for data disposal.

### Adverse event reporting and harms {22}

#### Adverse event reporting and harms

Risks greater than daily living incurred through participating in this trial are not anticipated. As a result, no discomfort or adverse events are anticipated for this study. However, in the study debrief form, participants will be provided with three potential routes for providing feedback (via the lead researcher), and/or for seeking support. Specifically, participants will read the following text: ‘If you have any issues that have arisen through participating in this study, you may wish to contact the short-term counselling and psychological therapies team based at (author institution) and you may wish to contact The Samaritans, a national helpline, which is a free, anonymous service on 116 123’. Where unexpected adverse events are reported to the researcher (or to the research institution), these will be logged and discussed with the primary institution’s Research Ethics Committee.

### Frequency and plans for auditing trial conduct {23}

Trial conduct will be audited within the remit of the Research and Governance Framework and Committee structure based at the primary research institution (UEL). Auditing of trial conduct is likely to occur on an annual basis by default.

### Plans for communicating important protocol amendments to relevant parties (e.g. trial participants, ethical committees) {25}

Details are provided in the ‘Plans for assessment and collection of outcomes {18a} section above.

### Dissemination plans {31a}

#### Dissemination plans

The results of project analyses will be published in English language peer-reviewed journals. Academic articles will be authored by the current protocol author and by two collaborators based at another UK-based Higher Education institution. Articles will be disseminated to key stakeholders (academics, public health policy makers and practitioners, non-governmental organizations who might make use of the face covering intervention) immediately following publication. In addition, a report containing study results using non-technical language will be produced to circulate to all relevant stakeholders. Contact details for requesting study results will be made available on study materials (i.e. the information sheet and debrief page). Trial materials will be available online via ClinicalTrials.gov. and in Supplementary Materials 1 and 2.

## Discussion

The purpose of the mental imagery intervention described in this protocol is, primarily, to increase adherence to a defined COVID-19 preventative behaviour (i.e. wearing face coverings on all occasions in public places where this is legally required). The secondary purpose of this protocol-defined intervention is to encourage more favourable intentions, attitudes, subjective norms, perceived behavioural control and to enhance barrier self-efficacy as a result of being allocated to the intervention (vs. allocation to a control condition). The broader aim is to develop the evidence base for behavioural science and behaviour change techniques applied to the context of COVID-19 preventative behaviours and COVID-19 preventative social cognitions in the interests of safeguarding public health from the harms posed by the spread of the virus.

Expert opinion from the Scientific Pandemic Influenza Group on Behaviours (SPI-B) has informed the UK's Scientific Advisory Group for Emergencies (SAGE) on how behavioural science can help encourage COVID-19 adherent behaviours among specific demographics. For example, a previous SPI-B report has highlighted the need for discrete interventions designed to target young people with behavioural adherence health messages focused on varied factors including informing/educating but also on presenting messages which ‘enable positive behaviours’ (e.g. how face coverings should be worn) [50]. A great advantage of mental imagery interventions is that they hold the possibility of strengthening links between thought and goal-directed action [51] potentially involving a neural basis whereby the rehearsal of actions helps foster more consistent subsequent behaviour [52]. The intervention described in this protocol is designed to target key theoretical determinants related to health-adherent behaviours involving beliefs/skills and will, therefore, also contribute to theoretical understanding through developing the evidence base relating to effective strategies to promote health behaviour change. Such evidence will form an important component part of efforts to understand how behavioural science can contribute to controlling the spread of COVID-19 both among UK university students and employees and, given the transmissible nature of the virus, among the general population and in target regions/demographics of concern.

Finally, we acknowledge the trade-offs involved in deciding on the focal parameters of the current study. A key strength of the study is that it adopts a factorial approach in which different kinds of mental imagery intervention—i.e. outcome imagery exercises, process imagery exercises, both in combination—will be tested in the context of adherence to wearing face coverings. Another strength is the range of theory-based social cognitive outcomes assessed in the intervention. By gauging this range of belief- and skill-based outcomes it will be possible to develop an understanding of the motivational pathways involved in successfully intervening in the context of COVID-19 preventative behaviours. A limitation of the planned study is the focus on a single behaviour change technique (BCT) intervention. There are many interventional strategies incorporating defined BCTs which would apply to promoting wearing face coverings as a defined COVID-19 preventative behaviour including implementation intentions [53], self-monitoring and providing opportunities for social comparison [54–56]. Aligned with commentary in the behavioural science literature it is also acknowledged here that wide ranging structural and cultural factors beyond intra-individual (e.g. motivational, dispositional) factors are involved with COVID-19 adherent behaviours including moral decision-making, leadership and stress and coping [57, 58]. On this basis, a mental imagery intervention could only realistically be employed as one component of a broader coalition of interventional approaches designed to address psychological, social and systemic/cultural factors. A further planned study limitation is the focus on individuals based solely at UK university campuses. In flagging this limitation we also note that UK university students might be understood as target population among whom face covering adherence should be prioritized; partly given the relatively high density of individuals within university campus spaces (which could drive COVID-19 transmission) and evidence of suboptimal COVID-19 protective measures among UK-based younger adults [59].

These areas of discussion notwithstanding, the paucity of any high-quality research in this area supports the focus on a single intervention in depth rather than multiple interventions in comparison at the current time. Should evidence support the mental imagery intervention described in this protocol, it will be important to apply the intervention to clustered COVID-19 preventative behaviour (e.g. wearing face coverings *and* social distancing *and* hand washing) and by comparing effects of different types of behaviour change interventional strategies.

## Trial status

Enrolment for this study began on 19 August 2020 and ended on 28 February 2021. The total number of participants recruited includes a sample with complete data at T1/T2 only (*n* = 465) and a sample with complete data at T1 and T3 (*n* = 297). This study protocol is Version 2.0, dated 9 September 2020.

## Supplementary Information

**Additional file 1: Supplementary Materials 1.** Mental Imagery Intervention Exercises To Promote Face Covering Adherence

**Additional file 2: Supplementary Materials 2.** Theory of Planned Behaviour & Barrier Self-efficacy Face Covering Adherence Measures
