# Supplementary material for: Mental imagery interventions to promote face covering use among UK university students and employees during the COVID-19 pandemic: study protocol for a randomized controlled trial
Source: Trials. 2022 Jan 18;23:51. doi: 10.1186/s13063-021-05852-y (PMC8764318; doi:10.1186/s13063-021-05852-y)
Supplement: Supplementary file 1 — Additional file 1: Supplementary Materials 1. Mental Imagery Intervention Exercises To Promote Face Covering Adherence [file 13063_2021_5852_MOESM1_ESM.docx]

**Outcome imagery exercise**

In the context of the coronavirus (COVID-19) outbreak, a face covering is something which safely covers the nose and mouth. A face covering can be reusable or single-use and can include any material that can securely fit round the side of the face. UK law requires individuals to wear face coverings on public transport, in shops and supermarkets (since 24th July 2020) and in enclosed public spaces where social distancing may be difficult or in public spaces where you come into contact with people you do not normally meet.

You are now asked to visualize yourself successfully wearing a face covering in all required public places/situations over the next week, and to imagine how you would feel. Imagine you have successfully managed to commit to this action and how satisfied you will feel. Imagine the planning and effort it has taken to do this. It is very important that you visualize yourself successfully wearing a face covering in all required public places/situations over the next week and to keep that picture on your mind.

Please write in the free-text box below how you imagine you will feel if you have successfully worn a face covering in all required public places/situations over the next week:

| <Participant writes in free-text box, unlimited length> |
| --- |

**Process imagery exercise**

In the context of the coronavirus (COVID-19) outbreak, a face covering is something which safely covers the nose and mouth. A face covering can be reusable or single-use and can include any material that can securely fit round the side of the face. UK law requires individuals to wear face coverings on public transport, in shops and supermarkets (since 24th July 2020) and in enclosed public spaces where social distancing may be difficult or in public spaces where you come into contact with people you do not normally meet.

You are now asked to visualize the kinds of strategies involved in successfully wearing a face covering in all required public places/situations over the next week. What personally accurate and meaningful strategies would help you successfully wear a face covering in all required public places/situations over the next week? Try to imagine the most effective strategies involved in successfully wearing a face covering in all required public places/situations over the next week. It is very important that you visualize yourself successfully wearing a face covering in all required public places/situations over the next week.

Please write in the free-text box below what kinds of strategies would be involved in successfully wearing a face covering in all required public places/situations over the next week:

| <Participant writes in free-text box, unlimited length> |
| --- |
