# Supplementary material for: Mental imagery interventions to promote face covering use among UK university students and employees during the COVID-19 pandemic: study protocol for a randomized controlled trial
Source: Trials. 2022 Jan 18;23:51. doi: 10.1186/s13063-021-05852-y (PMC8764318; doi:10.1186/s13063-021-05852-y)
Supplement: Supplementary file 2 — Additional file 2: Supplementary Materials 2. Theory of Planned Behaviour & Barrier Self-efficacy Face Covering Adherence Measures [file 13063_2021_5852_MOESM2_ESM.docx]

**Intention measure**

Over the next week, wearing a face covering while in public spaces where this is required* is something...

I intend to do

I plan to do

I will make an effort to do

Response options: 1=Strongly disagree, 5= Strongly agree

*on public transport; in shops and supermarkets; in enclosed public spaces where social distancing may be difficult; in public spaces where you come into contact with people you do not normally meet.

**Attitudes measure**

Wearing a face covering while in public spaces where this is required* over the next week is something…

Worthwhile–not worthwhile

Good–bad

Response options: 1=Important, 5=Unimportant

*on public transport; in shops and supermarkets; in enclosed public spaces where social distancing may be difficult; in public spaces where you come into contact with people you do not normally meet.

**Subjective norms measure**

Most people who are important to me (e.g., friends, family) would want me to wear a face covering while in public spaces where this is required* over the next week

Most people whose opinions I value would approve of my wearing a face covering while in public spaces where this is required* over the next week

Response options: 1=Strongly disagree, 5= Strongly agree

*on public transport; in shops and supermarkets; in enclosed public spaces where social distancing may be difficult; in public spaces where you come into contact with people you do not normally meet.

**Perceived behavioral control measure**

How much personal control do you think you have in wearing a face covering while in public spaces where this is required* over the next week

Response options: 1= No control at all, 5= Complete control

For me to wear a face covering while in public spaces where this is required* over the next week is

Response options: 1= Extremely difficult, 5= Extremely easy

Response options: 1= Impossible, 5= Possible

*on public transport; in shops and supermarkets; in enclosed public spaces where social distancing may be difficult; in public spaces where you come into contact with people you do not normally meet.

**Barrier self-efficacy measure**

[measure adapted from Hamilton et al. 2019]

Rate your degree of confidence in wearing a face covering while in public spaces where this is required* over the next week under the following conditions:

When I feel unhappy

When I feel stressed/frustrated

When I have a lot on my mind

When accessing my face covering is difficult

When wearing a face covering may feel uncomfortable (e.g. during warmer weather)

When others around me are not wearing face coverings

Response options: 1=Cannot do at all, 5= Highly certain can do

*on public transport; in shops and supermarkets; in enclosed public spaces where social distancing may be difficult; in public spaces where you come into contact with people you do not normally meet.

**Face covering adherence behaviour measure**

[measure adapted from Fisher et al. 2020]

In the past week, when you have gone outside your home for work, grocery shopping, or other activities that involved using public transport, visiting shops/supermarkets, being in enclosed public spaces where social distancing may be difficult, or being in public spaces where you came into contact with people do not normally meet, how often did you wear a cloth face covering^1^ that covered your nose and mouth?

Response options: 1=Never, 5=Always

^1^In the context of the coronavirus (COVID-19) outbreak, a face covering is something which safely covers the nose and mouth. You can buy reusable or single-use face coverings. You may also use a scarf, bandana, religious garment or hand-made cloth covering but these must securely fit round the side of the face. [material adapted from UK Government Cabinet Office (2020)]

References

Fisher, K. A., Barile, J. P., Guerin, R. J., Vanden Esschert, K. L., Jeffers, A., Tian, L. H., Garcia-Williams, A., Gurbaxani, B., Thompson, W. W., & Prue, C. E. (2020). Factors Associated with Cloth Face Covering Use Among Adults During the COVID-19 Pandemic—United States, April and May 2020. *MMWR. Morbidity and Mortality Weekly Report*, *69*(28), 933–937. <https://doi.org/10.15585/mmwr.mm6928e3>

Hamilton, K., Keech, J. J., Peden, A. E., & Hagger, M. S. (2019). Protocol for developing a mental imagery intervention: A randomised controlled trial testing a novel implementation imagery e-health intervention to change driver behaviour during floods. BMJ Open, 9(2). <https://doi.org/10.1136/bmjopen-2018-025565>

UK Government Cabinet Office. (2020). Face coverings: When to wear one and how to make your own. In: Rules and restrictions during coronavirus. https://www.gov.uk/government/publications/face-coverings-when-to-wear-one-and-how-to-make-your-own/
